# *CRY2* Is Associated with Rapid Cycling in Bipolar Disorder Patients

**DOI:** 10.1371/journal.pone.0012632

**Published:** 2010-09-09

**Authors:** Louise K. Sjöholm, Lena Backlund, Emarndeena Haji Cheteh, Inger Römer Ek, Louise Frisén, Martin Schalling, Urban Ösby, Catharina Lavebratt, Pernilla Nikamo

**Affiliations:** 1 Neurogenetics Unit, Department of Molecular Medicine and Surgery, Karolinska Institutet, Stockholm, Sweden; 2 Department of Clinical Neuroscience, Karolinska Institutet, Stockholm, Sweden; University of Texas Health Science Center, United States of America

## Abstract

**Background:**

Bipolar disorder patients often display abnormalities in circadian rhythm, and they are sensitive to irregular diurnal rhythms. CRY2 participates in the core clock that generates circadian rhythms. *CRY2* mRNA expression in blood mononuclear cells was recently shown to display a marked diurnal variation and to respond to total sleep deprivation in healthy human volunteers. It was also shown that bipolar patients in a depressive state had lower *CRY2* mRNA levels, nonresponsive to total sleep deprivation, compared to healthy controls, and that *CRY2* gene variation was associated with winter depression in both Swedish and Finnish cohorts.

**Principal Findings:**

Four *CRY2* SNPs spanning from intron 2 to downstream 3′UTR were analyzed for association to bipolar disorder type 1 (n = 497), bipolar disorder type 2 (n = 60) and bipolar disorder with the feature rapid cycling (n = 155) versus blood donors (n = 1044) in Sweden. Also, the rapid cycling cases were compared with bipolar disorder cases without rapid cycling (n = 422). The haplotype GGAC was underrepresented among rapid cycling cases versus controls and versus bipolar disorder cases without rapid cycling (OR = 0.7, P = 0.006−0.02), whereas overrepresentation among rapid cycling cases was seen for AAAC (OR = 1.3−1.4, P = 0.03−0.04) and AGGA (OR = 1.5, P = 0.05). The risk and protective *CRY2* haplotypes and their effect sizes were similar to those recently suggested to be associated with winter depression in Swedes.

**Conclusions:**

We propose that the circadian gene *CRY2* is associated with rapid cycling in bipolar disorder. This is the first time a clock gene is implicated in rapid cycling, and one of few findings showing a molecular discrimination between rapid cycling and other forms of bipolar disorder.

## Introduction

Chryptochrome 2 (CRY2) is a central component in the mammalian core circadian pathway that orchestrates rhythms that approximate the 24-hour day-night cycle [1]. Mood disorders often display disturbances in circadian rhythms that are manifested as abnormalities in sleep stage and various physiological domains, clinical efficacy of sleep deprivation [2], [3], and a molecular effect on the circadian clock by lithium [4], which is commonly prescribed for bipolar disorder. *CRY2* mRNA expression in blood mononuclear cells was recently shown to display a marked diurnal variation and response to total sleep deprivation in healthy human volunteers. It was also shown that bipolar patients in a depressive state had lower *CRY2* mRNA levels compared to healthy controls, and were nonresponsive to total sleep deprivation, and that *CRY2* gene variation was associated with winter depression in both Swedish and Finnish cohorts. The four-SNP risk and protective haplotypes spanned from *CRY2* intron 2 to downstream the 3′UTR and overlapped between the Swedish and the Finnish cohorts [5].

Bipolar disorder is characterized by episodes of the mood states mania/hypomania, depression, or mixed states at rates that vary markedly between and within individuals. Rapid cycling, defined according to DSM-IV as four or more mood episodes in any combination or order within *any* year in the course of the illness, is a severe and often treatment refractory form of bipolar disorder with tendency for more depressive than manic episodes [6], [7]. Rapid cycling has been associated to less favorable outcome with more pronounced functional impairment [8], [9], higher risk for suicide attempts and a higher rate of alcohol abuse [7]. Therefore, several researchers have tried to define rapid cycling as a distinct sub-group of bipolar disorder.

It is well known that bipolar patients are sensitive to irregular diurnal rhythms, and that a regular diurnal rhythm is one of the most important and effective focuses to reduce recurrence of bipolar episodes [10], [11]. Bipolar disorder cases are heterogeneous with regard to underlying genetic risk factors [12]. To possibly reduce the complexity of the genetic underpinnings we focused on rapid cycling, thus studying bipolar disorder patients with the highest recurrence risk, who might have a more vulnerable “diurnal system”. We tested the hypothesis that *CRY2* gene variation is associated with bipolar disorder, and rapid cycling in particular, in a Swedish bipolar cohort. The same four SNPs as analyzed previously [5] were analyzed in 557 bipolar cases and 1044 population controls.

## Results

The four *CRY2* SNPs rs10838524, rs7123390, rs10838527 and rs3824872, spanning from intron 2 to downstream 3′UTR, were analyzed for allelic association to bipolar disorder type 1, bipolar disorder type 2 and bipolar disorder with the feature rapid cycling versus population controls being anonymous blood donors (ABD). The A allele of rs10838524 was significantly overrepresented among bipolar disorder cases with rapid cycling compared to ABD controls (OR = 1.42, CI = 1.10−1.83, P = 0.0076), also after Bonferroni correction (cut-off for significance at P = 0.050/(3 case-control sets*2 SNP groups (defined by D'>0.80)) = 0.0083) (Table 1). To follow-up the association to rs10838524 a case-case design was performed. Rapid cycling bipolar cases (RC-BP) were compared with cases without rapid cycling (non-RC-BP). The results confirmed the association to rs10838524 (OR = 1.48, CI = 1.12−1.96, P = 0.0055) (Table 1). Neither bipolar disorder type 1 or bipolar disorder type 2 showed suggestive allelic association to any of the SNPs and were therefore not further analyzed (OR = 1.02−1.26 and 1.01−1.27, respectively, P>0.05).

**Table 1:** Allele frequency association results for rapid cycling.

SNP	Location	Alleles	Control set	MAF: A/U	OR [95% CI] a	P-value a	Empirical P-value
rs10838524	Intron 2	A*/G	ABD	0.52/0.44	1.42[1.10−1.83]	0.0076	0.022
			Non-RC-BP	0.52/0.43	1.48[1.12−1.96]	0.0055	0.018
rs7123390	Intron 8	A/G*	ABD	0.31/0.28	1.29[0.93−1.58]	0.16	0.26
			Non-RC-BP	0.31/0.26	1.29[0.96−1.74]	0.087	0.25
rs10838527	3′UTR	G/A*	ABD	0.11/0.079	1.51[0.98−2.33]	0.060	0.070
			Non-RC-BP	0.11/0.089	1.26[0.81−1.96]	0.31	0.075
rs3824872	Downstream 3′UTR	A/C*	ABD	0.23/0.21	1.51[0.86−1.56]	0.32	0.37
			Non-RC-BP	0.23/0.20	1.19[0.87−1.63]	0.27	0.36

Alleles, minor allele first.

*Ancestral allele in CEU population data (CEPH (Utah residents with ancestry from northern and western Europe)) from www.hapmap.org.

MAF, Minor allele frequencies for the affecteds (A) and unaffecteds (U).

ABD, anonymous blood donors.

Non-RC-BP, bipolar disorder patients without rapid cycling.

Odds ratio (OR), the proportion of minor versus major allele among affecteds (A)/proportion of minor versus major allele among unaffecteds (U).

a Logistic regression with gender as covariate was used.

Empirical *P*-value (EMP1), Point-wise *P-*value from 10,000 permutations.

There was a significant dosage effect (trend) in the association between the A allele of rs10838524 and rapid cycling (P = 0.0076) and this allele increased the risk for rapid cycling both in a homozygote and a heterozygote form (that is dominant model) compared to ABD controls (OR = 1.79, P = 0.0081) (Table 2). Also, rapid cycling cases compared to non-RC-BP showed a trend (P = 0.0041) and dominant model association (OR = 1.80, P = 0.0098) (Table 2). For the SNP rs3824872 there was no difference in allele frequency between rapid cycling cases and ABD controls or non-RC-BP, however the AA genotype was suggestively associated with a risk for rapid cycling in both the case-control and the case-case sample sets (OR = 2.16, P = 0.025 and OR = 2.38, P = 0.026, respectively). Likewise, rs10838527 G carriers were borderline overrepresented among cases compared to ABD controls (OR = 1.56, P = 0.046) (Table 2).

**Table 2:** Genotype association analysis results for rapid cycling.

SNP	Control set	Cases aa/ab/bb (%)	Cases *n*	Controls aa/ab/bb (%)	Controls *n*	Cochran-Armitage trend *P-* value^a^	P-value^a^ OR[95% CI]	
							Dominant model^b^	Recessive model^b^
rs10838524 A/G	ABD	25/54/21	145	20/50/31	914	0.0076	0.0081 1.79[1.16−2.75]	0.10 1.42[0.93−2.16]
	Non-RC-BP	25/54/21	145	18/50/33	396	0.0041	0.0098 1.80[1.15−2.85]	0.060 1.55[0.98−2.46]
rs7123390 A/G	ABD	11/40/49	146	9/38/53	928	0.16	0.23 1.24[0.87−1.78]	0.26 1.39[0.78−2.49]
	Non-RC-BP	11/40/49	146	6/39/55	398	0.12	0.24 1.26[0.86−1.84]	0.067 1.85[0.96−3.56]
rs10838527 G/A	ABD	0/22/78	147	0/15/84	945	0.039	0.046 1.56[1.01−2.42]	1.00
	Non-RC-BP	0/22/78	147	1/15/83	403	0.059	0.15 1.42[0.88−2.27]	1.00
rs3824872 A/C	ABD	9/28/63	146	4/33/63	943	0.32	0.86 1.03[0.71−1.49]	0.025 2.16[1.10−4.22]
	Non-RC-BP	8.9/28/63	146	4/32/64	400	0.26	0.77 1.06[0.72−1.57]	0.026 2.38[1.11−5.08]

ABD, anonymous blood donors.

Non-RC-BP, bipolar disorder patients without rapid cycling.

Alleles, minor allele (a) first.

a Logistic regression with gender as covariate was used.

b of minor allele.

Odds ratio (OR), the proportion of minor versus major allele among affected (A)/proportion of minor versus major allele among non-affected (U).

Pairwise linkage disequilibrium (LD) measure D' was 1.00 between all SNPs except between rs10838524 and rs7123390 where D' was 0.67. This overall high LD indicated that the SNPs spanning from rs10838524 to rs3824872 were tightly linked, thus all four SNPs were included in the haplotype block analyzed. Five haplotypes with frequency >1% were identified. The haplotype GGAC, including the protective alleles G from rs10838524, A from rs10838527 and C from rs3824872, was found less frequently in rapid cycling cases compared to ABD controls (OR = 0.75, P = 0.024) and compared to non-RC-BP (OR = 0.67, P = 0.0055) (Table 3). The haplotype AAAC, including the risk allele rs10838524 A was found suggestively more frequent in rapid cycling cases in both the case-control (OR = 1.34, P = 0.043) and in the case-case material (OR = 1.41, P = 0.033). Also, the haplotype AGGA was suggestively overrepresented in rapid cycling cases versus ABD controls (OR = 1.55, P = 0.048) (Table 3).

**Table 3:** Haplotype association analysis results for rapid cycling.

SNPs	Haplotype	Control set	Frequency cases	Frequency controls	OR^a^	P-value^a^
rs10838524-rs7123390-rs10838527-rs3824872	AGGA	ABD	0.11	0.078	1.55	0.048
		Non-RC-BP	0.11	0.090	1.26	0.30
	AGAA	ABD	0.12	0.13	0.94	0.74
		Non-RC-BP	0.12	0.11	1.1	0.65
	AAAC	ABD	0.28	0.23	1.34	0.043
		Non-RC-BP	0.28	0.22	1.41	0.033
	GAAC	ABD	0.038	0.047	0.80	0.49
		Non-RC-BP	0.039	0.039	0.95	0.88
	GGAC	ABD	0.45	0.51	0.75	0.024
		Non-RC-BP	0.45	0.54	0.67	0.0055

ABD, anonymous blood donors.

Non-RC-BP, bipolar disorder patients without rapid cycling.

Odds ratio (OR), the ratio specific haplotype versus all other haplotypes among the cases, relative to the ratio specific haplotype versus all other haplotypes among the controls.

a Logistic regression with gender as covariate was used.

## Discussion

This study gives evidence that the *CRY2* gene is associated with rapid cycling in bipolar disorder in Sweden. This is the first independent replication of the recent findings from gene expression analysis in white Americans that *CRY2* is associated to bipolar disorder, and from haplotype frequency analysis in Swedes and Finns that *CRY2* is associated to winter depression [5]. Hence, the present finding reinforces the significance of the previous reports implicating CRY2 in mood disorder in humans [5], and in rodent model [13].

The risk and protective *CRY2* haplotypes AGGA and GGAC, respectively, that were here indicated to be associated with rapid cycling in Swedish bipolar patients, were identical to those recently suggested to be associated with winter depression in Swedish patients, with similar case and control haplotype frequencies, and thereby similar effect sizes, between the two studies [5]. On single SNP-level, the A allele of the most upstream SNP studied, rs10838524, was associated with rapid cycling, similarly as with winter depression among Swedes. Similarly, the single-SNP results for rs10838527 and rs3824872 were in agreement between the two studies. This Swedish *CRY2* vulnerability locus overlaps with the reported Finnish winter depression vulnerability locus [5]. The rapid cycling risk and protective haplotypes AAAC and GGAC, respectively, were the opposite, that is protective and risk haplotypes, in the Finnish winter depression sample. This indicates allelic heterogeneity for the *CRY2* functional variation between the Swedish and Finnish populations.

Involvement of circadian gene variation in bipolar disorder is in agreement with the findings of circadian misalignments (i.e. the sleep-wake cycle is no longer in phase with the circadian rhythms) in patients with bipolar disorder [14]. Further, it is in agreement with the treatment efficacy of lithium targeting the circadian clock regulator glycogen synthase kinase 3β (GSK3β) [4], with findings of low melatonin levels in patients with bipolar and depressive disorder [15], and with increased melatonin suppression by light in bipolar disorder patients [16]. Melatonin appears to adjust the timing of circadian rhythm information, and has been suggested to be a marker for circadian rhythm phase and period [17]. In the circadian rhythm mainly phase advances (the location within a cycle at a particular time) has been reported in bipolar disorder, but also delays and reduced amplitudes (intensities of the oscillations) have been reported in mood disorder [18]–[20]. Therefore, the circadian misalignment may contribute to the pathogenesis of mood disorders. Both phase shifts and amplitude attenuations have been hypothesized to contribute to the pathogenesis [21].

Chryptochromes (CRY1 and CRY2) have a role in photoreception and in the core circadian clock itself. Biochemical and photochemical properties of human CRY2 have been described [22], and functional studies of Cry2 have been performed in rodents. However, the understanding of *CRY2* regulation and CRY2 function is still limited. Findings point to that CRY2 is activated in the evening [23]–[25], and that CRY2 is involved in the evening oscillator (light to dark transition) whereas CRY1 is involved in the morning oscillator [26]. In support, deletion of *Cry2* gene in mice prolonged the circadian period by approximately 48 min (from 23.7 to 24.5 hours) [27], and *Cry2* null mutants were more accelerated by light exposure than *Cry1* null mutants [28]. There are reports that support a disturbed evening oscillator, in line with a possible CRY2 dysfunction, in bipolar disorder type 1 [16] and winter depression [29], and there are data that indicate intact morning oscillator in patients with bipolar disorder [30] and in winter depression patients [31].

Lithium maintenance treatment is the drug of choice in especially bipolar disorder type 1, but rapid cycling patients often need a combination with valproic acid or other antiepileptic drugs. There are concerns about the risk to induce mood switches with antidepressant therapy. Therefore most guidelines recommend clinicians to avoid such medication for patients with rapid cycling. Currently, a major problem is the lack of tools to predict this form of illness. Medical treatment in our patients was clinically defined, thus no conclusions about treatment effects could be drawn.

The present finding, in particular in the case-case analysis, indicates that rapid cycling has some molecular distinction from bipolar disorder without rapid cycling. Several studies have tried to define rapid cycling as a distinct sub-group of bipolar disorder. Predictors such as family aggregation, early age at onset, and gender have been discussed, but the only consistent finding is a modest overrepresentation of women suffering from rapid cycling [7], [32]. From findings in molecular genetic association studies, there are some candidate genes for rapid cycling including *COMT* [33], *5-HTT* [34], [35] and *BDNF* [36], [37], but these findings are inconsistent and are in need for replication. An alternative to regard rapid cycling as a categorical dimension, is to consider the frequency of bipolar disorder episodes as a continuum where rapid cycling is a more severe course, which might be a result of a higher genetic loading. However, even though the *CRY2* SNPs rs10838524 and rs3824872 in our study appeared not to be associated with bipolar disorder type 1, we cannot exclude the possibility of a true association for in particular the *CRY2* SNPs rs7123390 and rs10838527 to bipolar disorder. Our inability to detect an association could reflect lack of power. Likewise, due to lack of power true single SNP association to rapid cycling for any of the SNPs without suggestive association cannot be excluded. Also, the bipolar disorder type 2 sample size was small. Furthermore, the anonymous blood donors (ABD) were population controls not screened for history of mental disorder. On the other hand, bipolar disorder cases are heterogeneous with regard to clinical symptoms and severity, and to genetic underpinnings [12]. This heterogeneity seems to pertain also to circadian rhythm abnormalities, exemplified by that melatonin suppression by light was seen in some but not other studies of bipolar disorder patients [16].

Circadian gene polymorphisms in *CLOCK* and *VIP* [38], [39], and *NR1D1*, *ARNTL* and *PER3* [40]–[43] have previously been associated to human bipolar disorder. Moreover, *ARNTL*, *RORA*, *RORB* and *RXRG* were associated with bipolar disorder in a meta-analysis integrating data from genome-wide association studies and human and animal model expression studies [44], [45]. *RORB* gene variation was found associated with bipolar disorder in children, which in general have a more rapidly cycling clinical presentation than adults [46]. Also, knock-out of *DBP* in mouse resulted in bipolar disorder with both phases [47].

Our patient material has not yet been analyzed for population stratification since only few genetic variations have been studied. However, a study of samples collected 2003 showed that the Swedish population has no strong internal genetic borders [48]. The vast majority of cases and controls in this *CRY2* study were living in Stockholm. In conclusion, this is the first report of genetic association between the circadian gene *CRY2* and a severe form of human bipolar disorder. It is also the first time a clock gene is implicated in rapid cycling, and one of few findings showing a molecular discrimination between rapid cycling and other forms of bipolar disorder. The finding is in line with a previous report on *CRY2* in human mood disorder, thus strengthening the evidence for the contribution of circadian gene variations to mood disorders.

## Materials and Methods

### Subjects and DNA

Written informed consent was obtained from the subjects. The study was approved by the Regional Ethical Review Board at Karolinska Institutet in Stockholm.

Patients (n = 577) were recruited from clinics in Stockholm County (n = 529), Southern Sweden (Skåne n = 41), and Northern Sweden (Västernorrland n = 7). They were diagnosed with bipolar disorder type 1 (n = 497, 43% men) or bipolar disorder type 2 (n = 60, 36% men), or bipolar disorder unknown type (n = 20). All medical records were studied, and focusing on the most severe manic episode the DSM-IV manic symptoms as well as rapid cycling and mixed episodes were registered. In order to complete the information participants were then interviewed over telephone by a psychiatrist or a special trained psychiatric nurse using the module for mania in Schedules for Clinical Assessment in Neuropsychiatry (SCAN) [49]. Individuals were excluded if mania was the result of alcohol or drug abuse, medication or somatic disease, as well as if close relatives were already included. The phenotypes rapid cycling (n = 155, 35% men), mixed episodes, the age of onset of mania as well as depression were registered. Population control DNA samples were collected from anonymous blood donors (ABD, n = 1044, 59% men) at Karolinska University Hospital, Stockholm, Sweden. Peripheral blood samples were collected and genomic DNA was extracted by SDS-urea by standard procedures and quantified by nano-drop. A previous study of samples collected in 2003 showed that the Swedish population had no strong internal genetic borders [48].

To analyze for genetic association to *CRY2,* a case-control design was applied: bipolar disorder type 1 versus ABD, bipolar disorder type 2 versus ABD, and bipolar disorder with rapid cycling versus ABD. In addition, an analysis using the case-case design was performed: bipolar disorder with rapid cycling versus bipolar disorder without rapid cycling (non-RC-BP, n = 422, 43% men) was used.

### Genotyping

We selected four SNPs in the *CRY2* gene: rs10838524, rs7123390, rs10838527 and rs3824872. These four SNPs have in a previous study been associated with vulnerability for depression [5]. All SNPs were genotyped on a 7900HT Fast Real-Time PCR System Instrument by using allele-specific Taqman MGB probes labeled with fluorescent dyes FAM and VIC (Applied Biosystems), according to manufacturer's protocols. Allelic discrimination was performed with the ABI PRISM 7900HT SDS and the SDS 2.2.1 program (Applied Biosystems).

### Statistical analyses

The three case-control sets and one follow-up case-case sample set were analyzed for allele frequency differences for the four *CRY2* SNPs using logistic regression with gender as covariate. Permutation analyses, with 10,000 permutations, were performed to obtain empirical significance values. Suggestive allele frequency difference (P<0.05) was found for rapid cycling versus ABD and rapid cycling versus non-RC-BP. These two sample sets were further analyzed for genotype and haplotype frequency differences, with gender used as covariate. P-values reported are uncorrected for multiple testing. P<0.05 was regarded as suggestively significant, whereas the Bonferroni corrected threshold P<0.0083 (0.050/(3 case-control sets*2 SNP groups (defined by D'>0.80))) was regarded as significant (Bonferroni correction considering the partial LD between markers [50], [51]). In the haplotype analysis all four SNPs were included since the pairwise linkage disequilibrium (LD), calculated between all four SNPs, were in strong LD. Allele, genotype and haplotype frequency difference tests as well as LD were calculated using the PLINK program, version 1.04 [52]. The trend tests with gender as covariate were performed in the Rcmdr package [53]. All four SNP fulfilled the criteria for Hardy-Weinberg equilibrium (HWE) among the controls (P>0.05). For genotyping quality control, 10% of the samples were genotyped twice and on average nine negative controls per 384 plate were used. The overall genotyping success was 91%. The power was ∼0.55 to detect an association between rapid cycling and rs10838524 for an allelic and a dominant model test, and between rapid cycling and rs3824872 for a recessive and a genotypic (2 *df*) model at á = 0.05 using ABD controls, whereas the power was lower for the other two SNPs. The power to detect association for bipolar disorder type 1 to rs10838524 and rs3824872 at above mentioned models, assuming similar effect size as found for rapid cycling, was >0.85 at á = 0.05, whereas it was far below 0.80 for the other two SNPs (http://pngu.mgh.harvard.edu/~purcell/gpc/cc2.html).
